# Manual Vagal Maneuver Effects on Cardiac Coherence, HRV, and Cognitive Performance in Young Healthy Women: A Pilot Study

**DOI:** 10.3390/ejihpe16020016

**Published:** 2026-01-26

**Authors:** Noemí SanMiguel, Clarys Custodio, Giada Aulicino, Miguel-Ángel Serrano

**Affiliations:** Department of Psychobiology, Universitat de València, 46010 Valencia, Spain; noemi.miguel@uv.es (N.S.); clarys_custodio@hotmail.com (C.C.); aulicino.2017794@studenti.uniroma1.it (G.A.)

**Keywords:** manual vagal maneuver, cardiac coherence, cognitive performance, attention, heart rate variability

## Abstract

Non-invasive vagus nerve stimulation (nVNS) is gaining attention as a promising approach to modulate emotional, cognitive, and autonomic processes. This exploratory study analyzed the short-term effects of manual vagal maneuver (MVM), applied to the left or the right side of the neck (carotid region), on emotional regulation, cognitive performance, and cardiac autonomic activity in healthy young females. Sixty participants, divided equally into three groups (left MVM, right MVM, and control), completed attentional tasks under their respective conditions. Heart rate variability (HRV), cardiac coherence, self-reported emotional states, and task performance were measured. The preliminary findings of this pilot study offer mixed evidence: while both stimulation groups seem to show significant improvements in attentional performance, only left-sided MVM was associated with increased cardiac coherence and elevated perceived emotional dominance. No significant changes were observed in HRV indices across groups, highlighting potential limitations of current physiological markers in capturing subtle autonomic modulation. These preliminary findings from a pilot study suggest that, in young healthy women, stimulation—particularly on the left side—may have a potential to enhance cognitive and affective functioning, even though no detectable changes were observed in conventional HRV metrics. Given the small sample size and other important methodological limitations, such as the single-session design, these results should be interpreted with caution, and replication in larger, more rigorous studies is necessary.

## 1. Introduction

The vagus nerve plays a key role in maintaining physiological homeostasis, by regulating reflex pathways that significantly influence heart function. As the primary neural structure of the parasympathetic branch of the autonomic nervous system (ANS), the vagus nerve is essential for controlling visceral functions in response to environmental stimuli. Persistent autonomic imbalances are widely recognized as precursors to various pathological conditions (Lathers & Schraeder, 2006; Rovere et al., 2003; Zuanetti et al., 1996).

Over the past decades, vagus nerve stimulation (VNS) techniques have emerged demonstrating effectiveness in managing a range of disorders. Invasive and non-invasive VNS techniques have demonstrated therapeutic benefits in pharmacoresistant epilepsy, depression, migraine, pain syndromes, and other neurological or neuropsychiatric disorders (Aboubakr et al., 2023; Bauer et al., 2016; Fang et al., 2016; Straube et al., 2015). Beyond clinical outcomes, non-invasive VNS (nVNS), including transcutaneous cervical and auricular stimulation, has also been associated with modulation of cognitive and emotional processes in healthy individuals. Reported effects include changes in memory performance, executive functioning, attentional engagement, and affective regulation, although findings remain heterogeneous and sometimes inconsistent across studies (Colzato & Beste, 2020; Tona et al., 2020; Zhang et al., 2022).

While most of the VNS literature focuses on electrical stimulation delivered via implanted or transcutaneous devices, alternative non-device-based approaches have also been proposed to modulate vagal activity. Among these, manual vagal maneuvers (MVM), such as carotid massage, aim to activate vagal reflexes primarily through stimulation of carotid baroreceptors. Carotid massage has long been used in clinical contexts to influence heart rate via baroreflex mechanisms and has been shown to effectively reduce heart rate in healthy young adults without inducing hypotension (Cheng et al., 2012; Green & Weaver, 2014; Schweitzer & Evan Teichholz, 1985). Despite conceptual similarities with VNS, manual maneuvers differ substantially in terms of stimulation specificity, controllability, and underlying physiological pathways.

In contrast to electrical VNS, empirical evidence regarding the short-term effects of manual vagal maneuvers on cognition and emotion remains scarce and fragmented. Most studies examining cognitive enhancement through vagal modulation rely on device-based stimulation, whereas manual approaches have received comparatively little systematic investigation, particularly in experimental settings and healthy populations (Abdullahi et al., 2024; Nonis et al., 2017). As a result, it is currently unclear whether MVM can induce measurable changes in cognitive performance or emotional regulation comparable to those reported for nVNS, or whether their effects are primarily limited to cardiovascular reflex modulation. This lack of evidence represents a relevant gap in the literature, especially given the simplicity, low cost, and accessibility of manual interventions.

From a mechanistic perspective, cognitive and emotional effects of VNS are generally attributed to the activation of afferent vagal fibers projecting to the nucleus of the solitary tract (NTS) and, subsequently, to limbic and cortical regions involved in emotion regulation and cognitive control. These pathways modulate key neurotransmitter systems, including noradrenergic and GABAergic signaling, which are known to influence cognitive performance (Colzato & Beste, 2020; Olsen et al., 2023). Although manual vagal maneuvers primarily activate vagal efferent pathways via baroreceptor reflexes, it has been hypothesized that secondary afferent signaling may contribute to downstream cognitive and emotional effects.

We hypothesized that vagal stimulation could influence psychological emotional measures and cognitive performance, possibly mediated by changes in heart rate variability (HRV), along with its effects on heart rate and cardiac coherence (CC). Heart rate variability (HRV) is widely used as a non-invasive marker of cardiac vagal modulation, with high-frequency HRV reflecting parasympathetic activity (Task Force of the European Society of Cardiology and the North American Society of Pacing and Electrophysiology, 1996). However, evidence regarding HRV changes following nVNS is mixed, with some studies reporting increases in parasympathetic indices and others finding null effects (Burger et al., 2020). Additionally, cardiac coherence (CC), reflecting synchronized oscillatory patterns across cardiovascular and respiratory systems, has been proposed as an additional exploratory marker of autonomic organization and attentional engagement (McCraty, 2022). Although CC is not a direct measure of vagal tone, it may capture autonomic states that support cognitive and emotional regulation under conditions of vagal modulation.

Consequently, the scientific evidence supporting manual vagal stimulation remains comparatively limited, particularly with respect to its short-term neuromodulatory effects on vagal reflexes and neurocognitive functioning (Abdullahi et al., 2024; Nonis et al., 2017). In this context, the present study was designed as an exploratory investigation to examine whether non-invasive vagal modulation via carotid massage (manual vagal maneuver, MVM) induces measurable psychophysiological changes—particularly in cardiac coherence (CC) and heart rate variability (HRV)—and whether such changes are associated with variations in emotional regulation and cognitive performance in healthy young women. Building on the broader literature on vagal modulation, the study compares the effects of left-sided, right-sided, and sham stimulation to explore potential laterality-dependent mechanisms. Given the anatomical asymmetry of cardiac vagal innervation, with the right vagus nerve predominantly influencing the sinoatrial node (Ahern et al., 2001), bilateral stimulation was applied to assess whether side-specific stimulation differentially affects autonomic and cognitive outcomes. We hypothesized that carotid massage could increase CC and HRV and, in turn, support attentional performance and emotional regulation.

## 2. Materials and Methods

### 2.1. Participants

Participants were recruited through announcements disseminated in various classes at the University of Valencia. Sixty-two women aged between 20 and 30 years were recruited (age: M = 21.98, SD = 1.61) and completed an online questionnaire to determine their eligibility based on pre-established inclusion and exclusion criteria. Exclusion criteria encompassed alcohol or substance abuse, smoking more than five cigarettes per day, the presence of cardiovascular, psychological, or neurological conditions, and the use of medications potentially affecting cardiac, emotional, or attentional functions (e.g., beta-blockers). Due to the very low number of male volunteers (fewer than five), which was insufficient to support meaningful statistical comparisons, and in order to enhance sample homogeneity and strengthen internal validity, the study was conducted exclusively with female participants. It is important to note that recruitment was performed randomly and without intentional selection bias; however, the resulting sample distribution was heavily skewed toward female participants due to differential rates of voluntary participation. This incidental gender imbalance, while unplanned, reflects common challenges in recruiting male volunteers for this type of study (Trauth et al., 2000) and was addressed by focusing the investigation on a more homogeneous group to reduce variability related to sex differences in autonomic regulation and cognitive performance.

### 2.2. Study Design

This study was intentionally conceived as a pilot investigation to assess the short-term feasibility and potential effects of MVM stimulation on emotional, autonomic, and cognitive parameters. A single-session design was selected to ensure experimental control and to minimize variability due to environmental and contextual factors, which is appropriate in early-stage research where the primary goal is to detect preliminary signals of efficacy and to refine procedures for larger trials. Participants were randomly assigned to one of three groups, and task administration was standardized across conditions to reduce confounding influences.

### 2.3. Procedure

Participants who met the eligibility criteria were invited to attend a laboratory session at the Faculty of Psychology, University of Valencia. Prior to the session, participants were instructed to adhere to their usual habits and sleep patterns, abstain from alcohol consumption, and avoid engaging in strenuous physical activity for at least 24 h before the experimental session. Additionally, participants were asked to refrain from smoking, consuming stimulant beverages or eating spicy food for at least 2 h before the experiment. The study received approval from the Ethics Research Committee of the University of Valencia and was conducted in accordance with the ethical principles outlined in the 1964 Declaration of Helsinki and its subsequent amendments. All participants received verbal and written information about the study and provided informed consent before beginning the experiment. The experiment was conducted in a single session lasting approximately one hour, scheduled either in the morning or afternoon. A single laboratory session was implemented to help maintain stable experimental conditions and focus on immediate physiological and psychological responses to stimulation. Given the specificity of the protocol and the intention to minimize potential sources of autonomic variability, this design was considered appropriate for assessing short-term effects. At the conclusion of each session, participants’ weight and height were measured and recorded. Therefore, each participant completed two identical testing blocks. Task 1 was administered as a baseline assessment. Immediately after completing Task 1, the manual vagal maneuver (MVM) was applied. Task 2 was then administered immediately after the MVM, with no additional instructions, as all task instructions had been provided prior to the first block.

### 2.4. Manual Non-Invasive Vagal Stimulation Procedure

Vagal stimulation was administered using a non-invasive technique known as carotid massage. This technique involves applying digital pressure to the carotid sinus. The procedure was as follows: the participant’s head was tilted approximately 20–30 degrees backward and slightly to the right to stimulate the reflex of left vagus nerve, and to the left to stimulate the reflex of right vagus nerve. The left carotid pulse was identified by palpation anterior to the sternocleidomastoid muscle, just below the angle of the jaw. Manual pressure was then applied to this region using the index and middle fingers of the right hand, in a posteromedial direction, for 15 s per application. Each application was followed by a 45 s recovery period. This procedure was repeated three times, resulting in three consecutive stimulation cycles (Cheng et al., 2012). Participants were divided into three groups: right reflex vagal stimulation (N = 21), left reflex vagal stimulation (N = 21), and a control group receiving a posterior neck massage without nervous system stimulation (N = 20).

### 2.5. Psychological and Performance Measures

#### 2.5.1. Emotional State

The Self-Assessment Manikin SAM; (M. M. Bradley & Lang, 1994) was used to assess emotional states before each task. The SAM is a pictorial self-report instrument that evaluates three main emotional dimensions: valence, arousal, and dominance. Each dimension is represented by a sequence of five humanoid figures, with the intensity of each dimension graded from positive to negative. For each dimension, a score of 1 represents the extreme of maximum pleasure (valence), greater activation (arousal), and a feeling of submission (dominance); up to 9, the extreme of maximum displeasure, relaxation and feeling of dominance, respectively. The SAM is culturally neutral and does not rely on language, making it suitable for diverse populations (Lang et al., 1990; Morris, 1995).

#### 2.5.2. Attentional Task

The “Letter Squares Test” (Cordero et al., 1990), a pen-and-paper task, was used to measure perception (stimulus discrimination) and sustained attention. Each participant received 90 matrixes of 16 letters (4 × 4) and another page of 50 matrixes if necessary. Participants had to find the repeated letter in a line or column. Participants identified the row or column with a repeated letter in a series of 4 × 4 letter matrices. After instructions and practice with six matrices, participants performed the task twice during the experiment (task 1 and task 2), each with a five-minute time limit. Performance was measured by the number of correctly completed matrices. This task was selected because it is an easy-to-administer measure of attention that was developed and validated in Spain by Cordero et al. (1990).

### 2.6. Electrophysiological Measures

#### 2.6.1. Cardiovascular Measures

Cardiovascular activity was recorded using the BIOPAC system (Biopac Systems, Inc., Goleta, CA, USA), which included the MP100 acquisition unit, the ECG100C electrocardiogram amplifier, and AcqKnowledge software version 4.2.0. Disposable pre-gelled electrodes (EL503) were connected via color-coded LEAD110 series cables and an MEC110C extension cable. ECG signals were acquired using Einthoven’s lead III configuration with three electrodes. Data were sampled at 1000 Hz and filtered using a digital band-pass FIR filter, with cut-off frequencies set at 1 Hz (low) and 35 Hz (high).

HRV analysis was conducted using Kubios HRV software (ver. 3.1) (Biomedical Signal Analysis Group, University of Kuopio, Finland; Tarvainen et al., 2014). Each ECG recording was visually inspected, and automatic artifact correction (low or medium level) was applied as needed. Participants were excluded if two or more segments were deemed unusable due to signal irregularities. In line with Task Force of the European Society of Cardiology and the North American Society of Pacing and Electrophysiology (1996), HRV was analyzed in multiple 5 min segments: one at baseline and two during each task condition.

To assess the impact of vagal stimulation on HRV, several frequency domain measures were chosen due to their strong empirical support and proven sensitivity to parasympathetic modulation, making them standard indicators in psychophysiological assessments of autonomic nervous system activity. In this sense, spectral power analysis was conducted using fast Fourier transformation (FFT), enabling the extraction of standard frequency bands: high frequency (HF; 0.15–0.40, low frequency (LF; 0.04–0.15 Hz), and very low frequency (VLF; 0.003–0.04 Hz), following established guidelines. Total HRV power (HRVtot) representing the accumulative power across all bands was also computed as a global index of autonomic variability (Liao et al., 2002; Svensson et al., 2016). The HF component is generally considered a specific index of parasympathetic control, whereas the LF band reflects both sympathetic and parasympathetic influences, particularly baroreflex engagement. HRVtot was used as a general indicator of autonomic variability. Together, these indices offer a multidimensional view of autonomic regulation and are sensitive to subtle shifts in vagal tone.

#### 2.6.2. Cardiac Coherence

Cardiac coherence was measured using emWave Pro, a biofeedback device by the HeartMath Institute designed for emotional regulation and stress management. This device includes an ear sensor, and a USB module connected to a computer running the emWave Pro software. The sensor was clipped to the participant’s earlobe, and heart rate data (intensity, rhythm, and RR intervals) were transmitted to the software. Logarithmic values called Avgcoherence measures were obtained for baseline, task 1, and task 2.

### 2.7. Statistical Analysis

Before conducting the primary analyses, the assumptions underlying parametric statistical tests were systematically evaluated. Normality was assessed using the Shapiro–Wilk test, sphericity was examined via Mauchly’s test, and homogeneity of variances was tested with Levene’s test. Given the small sample size and the violation of one or more of these assumptions across several variables, non-parametric statistical methods were deemed the most appropriate for subsequent analyses.

To analyze within-group differences between the two assessment moments (Moment 1 and Moment 2) across the three experimental groups (left MVM and right MVM, and control group), the Friedman test was applied. In addition, a Kruskal–Wallis test was used to assess baseline differences between the three experimental groups for CC and HRV_TOT_. This analysis was performed to ensure that groups did not differ significantly in these physiological parameters prior to the intervention. Neither test yielded statistically significant results: for cardiac coherence, χ^2^(2) = 5.572, *p* = 0.062; and for HRV_TOT_, χ^2^(2) = 5.177, *p* = 0.075. When the Friedman test indicated significant effects, post hoc analyses were conducted using the Wilcoxon signed-rank test to identify specific within-group changes over time. Kendall’s W was calculated as a measure of effect size for the Friedman test, with values of 0.1, 0.3, and 0.5 interpreted as small, moderate, and large effects, respectively. In cases where the Friedman test did not reach statistical significance, but Kendall’s W indicated at least a moderate effect size, pairwise comparisons were nonetheless performed using the Wilcoxon test to further explore potential differences between conditions. All analyses were conducted using SPSS 29.0 with an alpha level of 0.05.

## 3. Results

### 3.1. Attentional Task Performance

Concerning the attentional task, scores ranged from 11 to 43 in the first task (M = 23.29, SD = 6.47) and from 11 to 46 in the second (M = 26.06, SD = 8.84), suggesting a slight average improvement in task performance following MVM.

A Friedman test revealed significant overall differences between Moment 1 and Moment 2 across the three groups (χ^2^ = 9.931, *p* = 0.002). Kendall’s W = 0.40 indicates a moderate effect size. Significant differences between the experimental and control groups appeared: both the right (Z = −2.075, *p* = 0.038) and left (Z = −2.418, *p* = 0.016) MVM groups showed statistically significant increases in performance score, whereas the control group did not show any significant change (Z: −0.888; *p* = 0.375).

### 3.2. Emotional State

Valence scores ranged from 3 to 9 before MVM (M = 7.05, SD = 1.31) and from 2 to 9 afterward (M = 6.61, SD = 1.52). Arousal scores ranged from 1 to 9 in the first assessment (M = 4.21, SD = 1.72) and from 1 to 7 in the second (M = 3.87, SD = 1.61). For dominance, scores varied between 1 and 7 before MVM (M = 4.66, SD = 1.25) and between 2 and 9 after stimulation (M = 5.02, SD = 1.49).

In terms of valence, as presented in Table 1, a Friedman test revealed significant overall differences between Moment 1 and Moment 2 across the three experimental groups (χ^2^ = 4.500, *p* = 0.034). Kendall’s W = 0.73 indicates a large effect size, suggesting considerable variability across conditions. The Wilcoxon signed-rank test identified a significant decrease was observed in the control group (Z: −2.81; *p* = 0.005), reflecting a decline in positive emotional states. In contrast, no significant differences were observed in the left (Z: −0.256; *p* = 0.798) or right (Z: −0.714; *p* = 0.475) MVM groups. Regarding activation, no significant differences were observed between the two tasks across the three groups (χ^2^ = 4.500, *p* = 0.064), indicating stable levels of arousal. Kendall’s W = 0.55 indicates a moderate effect size, reflecting variability across conditions. Wilcoxon signed-rank test did not reveal significant changes in the control group (Z: −1.548; *p* = 0.122), the left (Z: −0.145; *p* = 0.885) or right (Z: −1.355; *p* = 0.176) MVM groups. No significant overall differences in dominance were observed across the three groups (χ^2^ = 2.500, *p* = 0.114). Kendall’s W = 0.40 indicated a moderate effect size, suggesting some degree of variability across conditions. Finally, the left MVM group exhibited a significant increase in dominance (Z: −2.124; *p* = 0.034), whereas no significant changes were found in the right MVM (Z: −0.771; *p* = 0.440) or control (Z: −0.096; *p* = 0.923) groups.

### 3.3. Cardiac Coherence

A Friedman test revealed no significant overall differences in CC between the two time points (χ^2^ = 2.323, *p* = 0.128). However, Kendall’s W = 0.37 suggested a moderate effect size, indicating some variability across conditions. Wilcoxon signed-rank test identified a significant difference across all participants (Z = −2.016; *p* = 0.044). As shown in Table 1, the left MVM group had a significant increase in CC (Z = −2.128; *p* = 0.033), while no significant changes were observed in the right MVM (Z = −0.191; *p* = 0.848) or the control group (Z = −1.130; *p* = 0.259).

### 3.4. Heart Rate Variability

For HRVtot, Friedman test revealed no significant overall differences between time 1 and time 2 across the three experimental groups (*p* > 0.05). When analyzed by group, only the control group showed a significant difference (Z: −2.017; *p* = 0.044), while no significant differences were found in the Right MVM group (Z = −2.0.75, *p* = 0.983) or the LeftMVM group (Z = −0.241; *p* = 0.809). Regarding LF, the Friedman test revealed no significant differences across the three groups (χ^2^ = 0.296, *p* = 0.586). For HF, although the Friedman test did not reach statistical significance (χ^2^ = 2.667, *p* = 0.102), Kendall’s W = 0.49 suggested a moderate effect size, justifying exploratory pairwise comparisons. The Wilcoxon signed-rank test revealed a significant difference across all participants (Z = −2.016; *p* = 0.044). As shown in Table 1, only the control group showed a significant decrease (Z: −2.154; *p* = 0.031), while no significant changes were observed in the right (Z: −0.065; *p* = 0.948) or the left (Z: −0.80; *p* = 0.936) MVM group.

**Figure 1:**
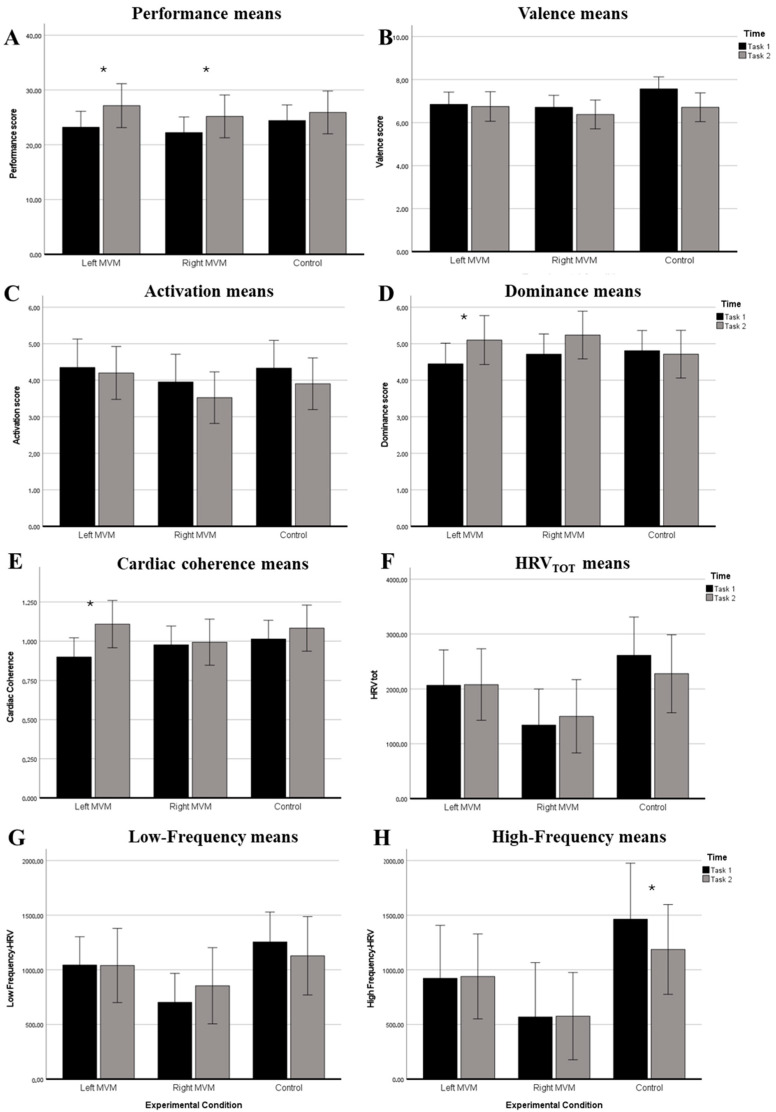
(**A**–**H**). Estimated marginal means by group and time point for each experimental variable. (**A**) Performance; (**B**) valence; (**C**) activation; (**D**) dominance; (**E**) cardiac coherence; (**F**) total HRV; (**G**) low-frequency HRV; (**H**) high-frequency HRV. Left and right MVM stimulation groups and the control group were assessed at two time points (Task 1 and Task 2). Bars represent estimated marginal means with error bars indicating the standard error of the mean. Within each group, Task 1 and Task 2 are displayed side-by-side for comparison. * *p* < 0.05, statistically significant difference between Task 1 and Task 2 within the same group.

To complement the information reported in Table 1, Figure 1A–H display the estimated marginal means by group and time point for each variable of interest.

## 4. Discussion

The present study offers mixed evidence regarding the short-term effects of MVM on emotional regulation, attentional performance, and cardiac autonomic activity in a sample of healthy young females. While both left and right MVM groups showed significant improvements in attentional task performance, only left-sided stimulation resulted in significant increases in CC and higher ratings of perceived dominance. However, HRV indices showed mixed and inconclusive results across groups, with patterns that were difficult to interpret clearly. Nevertheless, given the exploratory nature of this study and its methodological limitations, the results should be interpreted with caution. The discussion will therefore address the statistically significant findings observed, but it must be acknowledged that these do not necessarily reflect a clear or reliable real effect.

Although no significant HRV modulation was observed, the increase in CC in the left MVM group may suggest that CC could serve as a potentially more sensitive index of vagally mediated central-autonomic regulation; however, this interpretation should be considered preliminary given the study’s exploratory design and limitations. Prior studies have demonstrated associations between CC, vagal tone, and emotional regulation (R. T. Bradley et al., 2010; Laborde et al., 2022; Sevoz-Couche & Laborde, 2022; Vanderhasselt & Ottaviani, 2022). In line with this, our findings suggest a lateralized effect of vagal stimulation, with only left-sided MVM inducing significant physiological synchronization. While HRV indices such as HF and rMSSD are widely used markers of cardiac parasympathetic activity, these metrics have shown inconsistent sensitivity to non-invasive stimulation (Borges et al., 2019; Clancy et al., 2014; Sommer et al., 2023; Steenbergen et al., 2020). Only a minority of studies have observed increases in HF (Lamb et al., 2017; Tran et al., 2019) or rMSSD (Bretherton et al., 2019), and contradictory findings have been reported for LF components (De Couck et al., 2017; Gancheva et al., 2018).

The observed increase in CC following left MVM may reflect a shift toward greater physiological synchronization, as proposed by the Heart Rhythm Coherence Hypothesis (McCraty et al., 2009; McCraty & Shaffer, 2015). CC differs from traditional HRV metrics by capturing broader oscillatory patterns in autonomic regulation, often induced by paced breathing or parasympathetic entrainment. This state of physiological coherence has been linked to improved emotional stability and cognitive functioning (McCraty, 2022). However, the lack of control of respiration rate limits the ability to conclusively interpret cardiac coherence changes as reflecting vagal activity, since respiration can strongly influence CC measures. In our study, the enhancement of CC in the left MVM group was accompanied by increased dominance ratings which could reflect changes in perceived emotional control. Interpreted with caution, this increase in dominance may, over the long term, be associated with enhanced self-regulatory capacity in line with prior findings (McCraty & Shaffer, 2015; Tinello et al., 2023), although subjective arousal remained unchanged across groups.

Improvements in cognitive performance across both MVN groups suggest enhanced attentional processing, aligning with prior evidence that nVNS can modulate cognitive domains in healthy individuals. nVNS has demonstrated beneficial effects not only on various memory functions (Hassert et al., 2004; Jacobs et al., 2015; Konjusha et al., 2023; Zhang et al., 2022) but also on executive functions, including inhibitory control, cognitive flexibility, and attentional stability (Borges et al., 2019; Colzato & Beste, 2020). Our findings complement this body of work by providing preliminary evidence that manual stimulation techniques may similarly enhance attentional mechanisms. This would be consistent with reports of increased cognitive engagement and attentional control following nVNS interventions (Colzato & Beste, 2020; Jongkees et al., 2018). Nevertheless, it is important to contextualize these results within the broader, and sometimes inconsistent, literature. While many studies demonstrate cognitive benefits, others have reported null effects (Bömmer et al., 2024; Tona et al., 2020), possibly reflecting methodological differences in stimulation protocols, sample characteristics, or task demands. Given the exploratory nature of the present study and the small sample size, these findings should be interpreted cautiously. Future research with larger, more diverse samples and standardized stimulation parameters is needed to clarify the conditions under which mnVNS reliably enhances attention and other cognitive functions.

Moreover, the cognitive effects of MVM are likely mediated through the activation of vagal afferent fibers, which project to the NTS and subsequently influence higher-order cortical and subcortical regions involved in attention and cognitive control—modulating neurotransmitter systems such as noradrenaline and GABA (McCraty, 2022). This aligns with previous evidence showing improvements in memory and attention following afferent-focused VNS (Klaming et al., 2022; Ridgewell et al., 2021; Sommer et al., 2023; Zhang et al., 2022). The absence of concurrent HRV modulation in our study tempers conclusions regarding autonomic pathways and likely reflects the specific engagement of A-afferent fibers, which predominantly contribute to central neuromodulation while exerting limited influence on cardiac efferent outputs related to HRV (Yoo et al., 2013; Yuan & Silberstein, 2016).

These findings are also interpretable within the framework of the Heart Rhythm Coherence Hypothesis, which posits that beat-to-beat stability exerts top-down influences on emotion and cognition (R. T. Bradley et al., 2010; McCraty et al., 2009). The coherent cardiac state observed in the left mnVNS group may represent an optimized neurovisceral integration state, promoting attentional control (Bögge et al., 2022; Hansen et al., 2004), inhibitory function (Tinello et al., 2023), and memory performance (Zeng et al., 2023). According to the psychophysiological coherence model, such states enhance emotional resilience and cognitive efficiency, particularly when elicited through vagal afferent activation (McCraty & Shaffer, 2015; McCraty & Zayas, 2014).

The observed lateralization, where only left-sided MVM increased CC and perceived dominance, while attentional performance improved with both left and right stimulation, could suggest functional asymmetries in vagal anatomy. The right vagus nerve predominantly innervates the sinoatrial node and exerts stronger influence on heart rate (Ahern et al., 2001), while the left branch affects atrioventricular conduction and has broader connections to central autonomic and affective processing centers (Bremner et al., 2020). Indeed, the left vagus nerve has been more frequently associated with emotion-related brain networks, including projections to the nucleus tractus solitarius and the locus coeruleus, which are involved in mood and stress regulation (Nemeroff et al., 2006; Porges, 2007).

Moreover, the absence of HRV modulation aligns with prior findings on tVNS which similarly report null effects on traditional parasympathetic markers such as rMSSD and HF-HRV (Borges et al., 2019; Burger et al., 2019a, 2019b; Wolf et al., 2021), suggesting that these autonomic indices may also lack sensitivity to MVM induced changes. Several factors may account for this, including the fact that cardiac autonomic modulation depends on activation of B-efferent fibers, which require higher stimulation thresholds than those typically achieved with non-invasive techniques (Yoo et al., 2013). Furthermore, individual differences in baseline vagal tone, posture during stimulation, and stimulation parameters (e.g., frequency, intensity, duration) can all influence autonomic responses.

In conclusion, the findings suggest that left-sided MVM could elicit specific psychophysiological modifications. Furthermore, attentional performance improvements observed in both left and right stimulation groups indicate that manual non-invasive vagal stimulation, irrespective of lateralization, may facilitate cognitive enhancement. However, the lack of consistent HRV changes highlights the need to consider complementary markers such as CC when assessing the autonomic and cognitive effects of non-invasive vagal stimulation.

### Limitations

This study presents several limitations that warrant consideration. First, the small sample size restricts the generalizability of the findings and increases the risk of Type II errors, while also limiting the statistical robustness of post hoc comparisons and precluding the application of corrections for multiple testing without increasing the likelihood of false negatives. The exclusive inclusion of healthy young female participants further limits the applicability of the results to other populations, such as males, older adults, or clinical groups. Although this gender imbalance reflects low male participation during recruitment, its statistical improbability suggests systematic bias and should be addressed in future research with more balanced and representative samples. Moreover, we did not assess participants’ habitual physical activity or exercise routines, which could influence baseline vagal tone and responsiveness to MVM. Future studies should include physical activity measures to control this factor.

Second, the absence of a fully credible sham condition and the lack of blinding introduce a risk of expectancy and experimenter effects, particularly in self-reported measures such as affective ratings. The control procedure (posterior neck massage) did not match the experimental condition in sensory experience or perceived plausibility, which may have influenced group differences in emotional outcomes. Furthermore, while the stimulation targeted the carotid sinus region, the vagal specificity of manual MVM remains uncertain, especially given the lack of quantification of pressure, anatomical precision, or physiological verification. The observed changes cannot be conclusively attributed to vagal engagement. The short-term, single-session design further limits our understanding of the potential sustained or cumulative effects of MVM, as no follow-up assessments were included. While this format allowed for tight control over environmental and procedural variables, it necessarily limits the scope of inference to immediate post-stimulation responses, precluding conclusions about longer-term physiological or cognitive changes. Though the timing of sessions (morning vs. afternoon) was evenly distributed across groups, circadian variability may have influenced physiological or affective responses.

Another key limitation concerns the use of CC as a physiological outcome. Although CC was included as an exploratory marker due to its proposed link to attentional regulation and vagal activity, it is highly sensitive to respiration, which was not controlled, and cannot be assumed to more accurately reflect vagal tone than standard HRV indices. Notably, no significant group differences were observed across HRV parameters, challenging theoretical claims that HRV mediates the effects of vagal stimulation on cognition. Relatedly, the interpretation of increased subjective “dominance” in one stimulation group should be treated with caution, as the SAM scale measures perceived emotional control and does not directly assess executive functioning or agency. Methodological heterogeneity across studies also complicates interpretation and comparison of findings. As Borges et al. (2019) note, the efficacy of tVNS may not depend strictly on stimulation intensity, but rather on nonspecific neural signals that reach brainstem regions and trigger generalized modulatory effects. This perspective underscores the need for caution when comparing stimulation protocols and interpreting physiological outcomes. Taken together, these limitations emphasize the exploratory nature of the study and the need for future research using validated stimulation protocols, stronger experimental control, longitudinal follow-up, and larger, more diverse samples.

Finally, although the protocol was conducted in a seated position to enhance ecological validity—reflecting real-life applications of MVM during everyday activities (Antonino et al., 2017)—posture itself is known to influence autonomic regulation (Watanabe et al., 2007). This may partially account for discrepancies between our findings and those from studies using supine or semi-supine positions.

## 5. Conclusions

In conclusion, given the pilot nature of the study, these results should not be interpreted as evidence of causal effects; however, they indicate that MLVM is related to cognitive performance and emotional dominance in the present sample. These findings must be interpreted with caution given the small sample size, single-session design, and the absence of significant changes in HRV, which highlight both the complexity of vagal pathways and the limitations of current physiological markers in detecting subtle autonomic effects. Future studies with larger, more diverse samples, refined stimulation protocols, and multimodal assessments are needed to clarify the robustness, mechanisms, and possible clinical relevance of these effects. At this stage, MVM should be considered a promising but unconfirmed approach whose potential applications require more rigorous investigation.

## Figures and Tables

**Table 1:** Means and standard deviations of measures.

	Left MVM (N = 21, M = 21.85 ± 1.63)		Right MVM (N = 21, M = 22.14 ± 1.45)		Control (N = 20, M = 21.95 ± 1.80)	
	Task 1	Task 2	Task 1	Task 2	Task 1	Task 2
Performance	**23.2 (6.96)**	**27.15 (8.85)**	**22.24 (6.19)**	**25.19 (8.91)**	24.43 (6.38)	25.9 (9.08)
Emotional State						
Valence	6.85 (1.66)	6.76 (1.41)	6.71 (1.27)	6.38 (1.77)	**7.57 (0.75)**	**6.71 (1.38)**
Activation	4.35 (1.90)	4.2 (1.61)	3.95 (1.77)	3.53 (1.50)	4.33 (1.53)	3.9 (1.73)
Dominance	**4.45 (1.19)**	**5.1 (1.33)**	4.71 (1.31)	5.24 (1.67)	4.81 (1.29)	4.71 (1.45)
CC	**0.90 (0.26)**	**1.11 (0.34)**	0.98 (0.28)	0.99 (0.28)	1.01 (0.29)	1.08 (0.39)
HRV_TOT_	2068.10 (1342.86)	2080.45 (1257.53)	1343.10 (915.63)	1502.56 (1231.51)	2612.19 (1819.00)	2277.24 (1740.31)
LF	1044.31 (544.04)	1040.03 (634.99)	702.62 (502.61)	854.73 (888.55)	1255.73 (633.19)	1128.87 (660.03)
HF	922.85 (934.80)	939.74 (850.03)	568.68 (441.63)	576.38 (461.45)	**1463.73 (1527.65)**	**1186.60 (1107.55)**

Note: Bold values indicate significant changes between the two measures. CC = Cardiac Coherence; HRV_TOT_ = Total Heart Rate Variability; HR = Heart Rate; LF = Low Frequency; HF = High Frequency. Values are expressed as mean (SD). Higher scores indicate stronger feelings in the emotional state.

## Data Availability

The raw data supporting the conclusions of this article will be made available by the authors on request.
